# Factors Regulating or Regulated by Myogenic Regulatory Factors in Skeletal Muscle Stem Cells

**DOI:** 10.3390/cells11091493

**Published:** 2022-04-29

**Authors:** Tomohiko Shirakawa, Takashi Toyono, Asako Inoue, Takuma Matsubara, Tatsuo Kawamoto, Shoichiro Kokabu

**Affiliations:** 1 Division of Orofacial Functions and Orthodontics, Department of Health Improvement, Kyushu Dental University, Kitakyushu 803-8580, Japan; r16shirakawa@fa.kyu-dent.ac.jp (T.S.); asako120120@gmail.com (A.I.); r15kawamoto@fa.kyu-dent.ac.jp (T.K.); 2 Division of Molecular Signaling and Biochemistry, Department of Health Improvement, Kyushu Dental University, Kitakyushu 803-8580, Japan; r15matsubara@fa.kyu-dent.ac.jp; 3 Division of Anatomy, Department of Health Promotion, Kyushu Dental University, Kitakyushu 803-8580, Japan; toyono@kyu-dent.ac.jp

**Keywords:** satellite cell, myogenesis, MyoD, TLE3, taste receptor, NF-κB, p65

## Abstract

MyoD, Myf5, myogenin, and MRF4 (also known as Myf6 or herculin) are myogenic regulatory factors (MRFs). MRFs are regarded as master transcription factors that are upregulated during myogenesis and influence stem cells to differentiate into myogenic lineage cells. In this review, we summarize MRFs, their regulatory factors, such as TLE3, NF-κB, and MRF target genes, including non-myogenic genes such as taste receptors. Understanding the function of MRFs and the physiology or pathology of satellite cells will contribute to the development of cell therapy and drug discovery for muscle-related diseases.

## 1. Introduction

The skeletal muscle accounts for 40% of the total body weight, and the metabolism and motor functions of skeletal muscles are essential for human life. Loss of skeletal muscle mass leads to various negative effects, including obesity, insulin resistance, diabetes, decreased quality of life that may require dependence on others and hospital stays, and mortality [1]. As the number of elderly people in society is increasing, it is important to establish strategies for improving skeletal muscle mass and function in aged individuals. Therefore, understanding the physiology and pathology of satellite cells, such as skeletal muscle stem cells, is essential.

During skeletal muscle generation, known as myogenesis, myoblasts fuse with myofibers. Myogenesis is divided into two distinct phases [2]. In the embryonic stage, the structures derived from the mesoderm generate body muscle fibers; these fibers act as a template for the generation of additional fibers [3,4]. In the perinatal stage, myogenic progenitors residing in muscles initially proliferate extensively. Subsequently, the number of myonuclei reaches a steady state, and the protein synthesis in myofibers reaches a peak [5,6]. Once the muscle matures, these progenitors enter a period of quiescence and exist between the muscle fibers and basal lamina as satellite cells. In the adult stage, myogenesis depends on satellite cells to maintain tissue homeostasis, similar to in other organs that undergo renewal [7,8,9].

MyoD, Myf5, myogenin, and MRF4 (also known as Myf6 or herculin) are myogenic regulatory factors (MRFs), which act as master transcription factors that are upregulated during myogenesis and influence stem cells to differentiate into myogenic lineage cells [10,11,12,13]. MRFs regulate myogenesis by modulating the expression of both myogenic and non-myogenic genes. However, the mechanisms by which MRFs regulate non-myogenic genes remain unknown. Although signaling molecules mediate myogenesis and MRFs, these signaling mechanisms are not completely understood. In this review, we summarize MRFs and focus on their regulatory factors, such as transducin-like enhancer of Split3 (TLE3), nuclear factor-kappa B (NF-κB), and MRF target genes, including non-myogenic genes such as taste receptors.

## 2. Muscle Stem Cells and MyoD

Satellite cells are typically quiescent during the cell cycle; however, when these are activated by stimulation, such as by muscle injury, the expression of MRFs is upregulated and satellite cell progenitors commit to the myoblast lineage and proliferate rapidly [14,15]. Satellite cells are abundant during muscle regeneration and postnatal muscle growth [16]. In addition to satellite cells, mesangioblasts [17], Pw1^+^/Pax7^+^ interstitial cells [18], and TWIST2^+^ progenitors [19] are present in the population of muscle stem cells and can be directly converted to myogenic lineage cells. Although these cell classifications do not conform to the current characterization and many of these cells have overlapping markers and functions, the expression of MyoD is upregulated in all of these cells during myogenesis [20,21,22]. MyoD was first identified by the Harold M. Weintraub group. MyoD was cloned during a functional analysis of muscle fiber formation, and the expression of cDNA for the mouse MyoD protein converted murine embryonic fibroblast C3H10T1/2 into myogenic cells [22]. Ectopic expression of MyoD can also induce human induced pluripotent stem (iPS) cells or murine embryonic stem cells to differentiate into myogenic cells [23,24,25,26,27]. Therefore, introducing MyoD into iPS cells shows potential as a safe and effective approach for regenerative medicine applications in the muscle.

## 3. Structure and Function of MRFs

**Figure 1:**
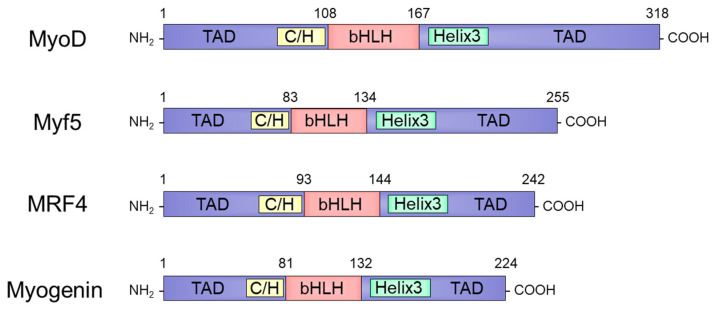
Protein structure of myogenic regulatory factors.

MyoD is an MRF transcription factor, along with Myf5 [10], myogenin [11], and MRF4 [12,13]. Despite variations in their lengths and amino acid sequences, each MRF is composed of three domain structures, including a highly conserved basic helix-loop-helix (bHLH) domain with myogenic potential [28]. This bHLH domain is flanked by an N-terminal region with a C/H domain and C-terminal region with a helix 3 domain that mediates the transcriptional region [29]. Phylogenetic analyses of the amino acid sequences of murine MRFs showed that the similarity between MyoD and Myf5 is 53%. The similarities between myogenin and MyoD and Myf5 are 38% and 40%, respectively. The similarities of MRF4 with MyoD and Myf5 are 43% and 40%, respectively (Figure 1) [30]. MRFs exhibit functional redundancy. Each MRF can induce myogenesis and is anthropogenically expressed in various non-myogenic cells such as fibroblasts, adipose, nerve, and liver cells, indicating a functional overlap in establishing the muscle cell lineage [31]. Experiments using MRF-null mice revealed hierarchical relations and overlapping functions as features of the regulatory network of MRFs [32]. Moreover, newborn mice null for both MyoD and Myf5 were completely deficient in myoblasts and myofibers. MyoD and Myf5 are essential for myogenic lineage cell commitment and act upstream of MRF4 and myogenin [12,33,34]. Further experiments focusing on these differences are required to understand the detailed functions of each MRF.

## 4. Role of MRFs during Myogenesis after Birth

**Figure 2:**
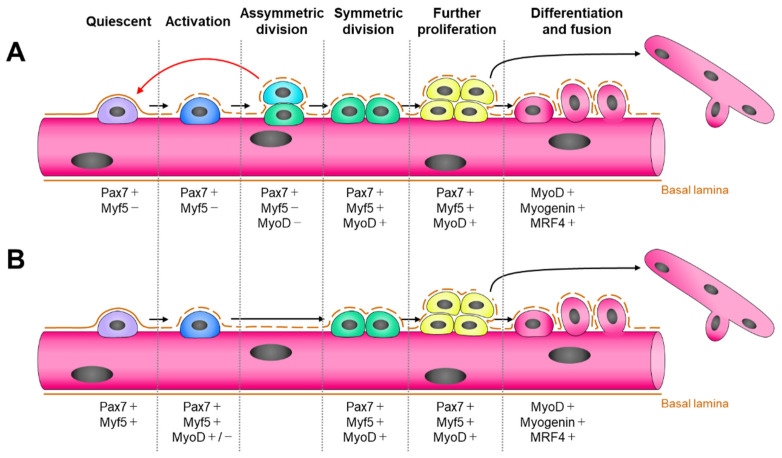
Expression of myogenic regulatory factors during different developmental stages of satellite cells. Cells can divide into two groups, myogenic stem cells (Pax7^+^/Myf5^−^) (**A**) and myogenic precursor cells (Pax7^+^/Myf5^+^) (**B**). The figures are referred from the review paper by Asfour et al. [40].

Paired box gene 3 (Pax3) and paired box gene 7 (Pax7) are transcription factors. In postnatal muscle cells, Pax3 and Pax7 are markers of muscle stem cells that reside beneath the basal lamina of mature myofibers [35]. Pax7 is expressed in all satellite cells of myofibers after birth, whereas Pax3 is not. Chromatin immunoprecipitation (ChIP) sequencing of primary myoblasts revealed that although Pax3 and Pax7 recognize the same DNA motifs, Pax3 binds with a lower affinity compared to that of Pax7. Pax3 binds to a subset of Pax7 target genes that mainly regulate embryonic function and maintain undifferentiated satellite cells. In contrast, Pax7 activates the expression of genes involved in maintaining the postnatal satellite cell phenotype by regulating proliferation and inhibiting differentiation [36]. Satellite cells are typically quiescent, meaning that they are normally arrested in the G0 phase of the cell cycle [37]. During this phase, the cells can be divided into two groups, myogenic stem cells (~10%, Pax7^+^/Myf5^−^) [38] and myogenic precursor cells (~90%, Pax7^+^/Myf5^+^) [39], based on their self-renewal ability (Figure 2) [40]. When satellite cells are activated by stimuli such as muscle injury, the expression of Pax7 [21] and Pax3 [41] gradually decreases in most satellite cells. In contrast, muscle satellite cells begin to increase MRF expression to ensure a myogenic program [42]. Myogenic stem cells (Pax7^+^/Myf5^−^) exhibit asymmetric division, during which some daughter cells return to myogenic stem cells (Pax7^+^/Myf5^−^) to serve as a stem cell reserve. Myogenic precursor cells (Pax7^+^/Myf5^+^) exhibit symmetrical division, in which every daughter cell is committed to the myoblast fate and begins to differentiate [39]. In addition to the expression of Myf5 and Pax7, satellite cells begin to express MyoD protein during the early stages of activation [43]. Pax7 is necessary to activate satellite cells by directly binding to the promoter and enhancer regions of *MyoD* and *Myf5*, respectively [36]. The basal lamina remnants guide activated satellite cell progeny that is committed to myogenesis to migrate and proliferate. Increased MyoD expression with MRF4 and myogenin expression stimulates the differentiation of these cells. Terminally differentiated satellite cells may fuse into either newly formed myofibers or pre-existing myofibers (Figure 2) [40]. The main function of MyoD is to withdraw cells from the cell cycle by enhancing the expression of myogenin and p21. p21 inhibits cyclin-dependent kinases, which inhibit MyoD. Therefore, MyoD expression enhances MyoD activity in satellite cells in a feedback and/or feedforward manner. Continuous expression of MyoD is required to sustain muscle-related gene expression [44] and is a critical effector of the fast-twitch muscle fiber phenotype (type IIA, IIB, and IIX) [45,46].

## 5. Transcriptional Mechanism of MRFs

MRFs dimerize with other HLH-containing proteins through HLH–HLH interactions [47]. MRFs also form heterodimers with E proteins, including E47 and E12, through the bHLH domain and can bind to the E-box sequence motif containing the nucleotide CANNTG. Therefore, heterodimer formation activates transcription at the promoters of muscle-specific genes [48]. MRFs are thought to bind to hundreds of muscle-related gene promoters to result in stem cell myogenesis. Differences in the functions of MRF are related to their ability to induce different programs of gene expression. Studies are needed to understand how MRFs bind to the genome during skeletal muscle regeneration and development. The binding dynamics of MyoD have been extensively studied using cultured cells with high-throughput technologies, including DNA ChIP sequencing [49,50,51,52]. In vitro experiments using C2C12 cells revealed the MyoD-binding site during myoblast differentiation [53].

In addition to the regulatory elements of genes that are upregulated or downregulated during myoblast differentiation, MyoD recognizes and binds to a large number of E-box sites that are not related to myogenesis. Cao et al. compared the number of binding sites in myoblasts and myotubes and identified 23,000 and 26,000 MyoD binding sites in myoblasts and myotubes, respectively; they also showed that MyoD binding is more stable after differentiation. MyoD binding is correlated with regional histone acetylation rather than with local histone acetylation [53]. The biological importance and functions of these sites remain unclear because most of these sites are inactive when functional enhancer tests are performed in vitro. Mousavi et al. confirmed that the amount of bound MyoD in myotubes is higher than that in myoblasts (39,700 and 18,142, respectively) [54]. Both analyses revealed a discrepancy in the number of binding sites [53,54]. These differences are likely related to differences in the methods used to identify the peaks and boundaries of compartmentalization reading in the experiments. Using RNA-sequencing analysis, Mousavi et al. revealed that both MyoD and myogenin share most of the 35,000 binding sites [54]. In myotubes, these sites are also bound by polymerase II and are marked by H3K27Ac and H3K4me1 [54]. A total of 1053 Myf5 sites were identified in myoblasts, and 9300 and 1428 MyoD binding sites were identified in myotubes and myoblasts, respectively. The overlap between the binding sites in myoblasts is 30% [52].

To evaluate the dramatic differences in the number of MyoD-binding sites between myotubes and myoblasts, we focused on transcriptional repressors targeting the same DNA-binding motif as HLH proteins, including MRFs. Snail1 and Snail2 are expressed in myoblast lineage cells and repress transcription by recruiting histone deacetylases 1 and 2 [55,56,57]. Snail1 binds to E-boxes containing a G/C-rich nucleotide associated with genes expressed almost exclusively in myotubes. In contrast, Snail2 does not bind to E-boxes with A/T-rich nucleotides associated with gene expression in myoblasts, suggesting that Snail must be removed for MyoD to access the genes expressed in the myotube. The expression of Snail is also regulated by MRFs. MRFs induce the expression of miRNA-30 and miRNA-206, which suppress Snail1/2 expression in myotubes. Taken together, downregulation of Snail1/2 allows MyoD to access its binding sites [52].

## 6. Target Genes of MRFs

**Figure 3:**
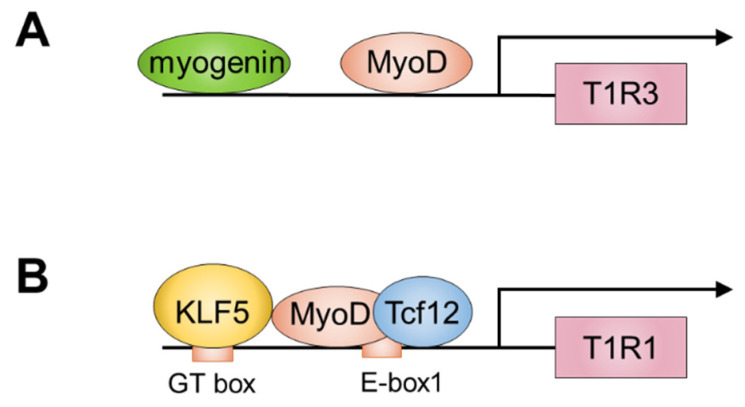
Myogenic regulatory factors regulate the expression levels of T1R1 and T1R3 during myogenesis. MyoD and myogenin binding the promoter region of T1R3 (**A**). The heterodimer of MyoD and Tcf12 interacts with Klf5 and binds to promoter region of T1R1 (**B**).

Taste is a guide and guardian of food intake and is necessary for maintaining human health [58]. Taste can be divided into five categories: salty, sweet, umami, sour, and bitter [59]. The salty, sweet, and umami tastes encourage the consumption of foods containing minerals, carbohydrates, and amino or nucleic acids, respectively, whereas sour and bitter tastes prevent ingestion of strong acids and potentially toxic substances, respectively [59]. The taste 1 receptor (T1R) family belongs to the G-protein-coupled receptor family and is composed of three members: T1R1, T1R2, and T1R3. These receptors form heterodimers with each other and recognize different ligands depending on the combination of receptors. T1R3 forms a dimer with T1R2 to act as the sweet taste receptor that responds to molecules, such as sugars, whereas T1R3 complexes with T1R1 form the umami taste receptor that responds to nuclear acids and amino acids [60,61,62,63]. Although these functions were originally thought to occur in the gustatory tissue inside the mouth, the first study showing that T1Rs and molecules involved in taste signal transduction are present in the stomach and intestine was reported in the late 1990s [64]. Subsequently, many researchers reported the expression and functional roles of T1R family members in various tissues and cell types, including in the adipose tissue [65], pancreatic β-cells [66], central nervous system [67], and heart [68]. Therefore, despite the name “taste receptor,” these G-protein-coupled receptors are now recognized as “nutrient sensors” with critical roles in chemoreception for detecting amino acids and glucose status [69]. We also reported the function of both T1R3 and T1R1, which are components of the umami receptor in the skeletal muscle [70,71,72]. The expression of T1R1 and T1R3 is regulated by MRFs [70,71,72]. ChIP analysis revealed that both MyoD and myogenin bind to the promoter region of T1R3 (Figure 3A). We also demonstrated that MyoD induces the promoter activity of T1R3, as assessed in a luciferase reporter assay. Comparative genomic analysis revealed that the gathering regions of MyoD-and myogenin-binding sites are highly conserved across species [70]. These observations are consistent with the fact that T1R3 expression increases with myogenic differentiation in vitro [70].

MyoD regulates the expression of T1R1 in both myoblasts and myotubes during myogenesis [50]. We determined the mechanism of the regulation of T1R1 expression through T1R1. Krüppel-like factor 5 (Klf5), a zinc finger protein, acts as a transcription factor [73] that regulates the transactivation of T1R1 during myoblast differentiation in C2C12 cells as Klf5 binds to the GT box (CCACCC) in the promoter region of T1R1 [71]. Klf5 also directly binds to MyoD and regulates muscle-specific genes associated with myoblast differentiation [74]. Based on these findings, we assessed the role of Klf5 in the transactivation of T1R1 in combination with MyoD. We examined the relationship between Klf5 and MyoD in regulating the gene expression of T1R1 during myoblast differentiation of C2C12 cells. Our results showed that MyoD binds to the T1R1 promoter region, which has three E-boxes (E-box 1–3). The heterodimer of MyoD and Tcf12 interacts with Klf5 and binds to E-box 1, activates transcription, and increases the expression levels of T1R1 during myoblast differentiation [72] (Figure 3B).

These findings agree with those of studies showing that T1R3 and T1R1 are highly expressed in the skeletal muscles. Skeletal muscle cells from T1R3-null mice exhibit impaired mammalian targets of rapamycin complex 1 signaling and an increase in autophagy [69,75,76]. This suggests that T1R3 forms a heterodimer with T1R1 as an umami receptor and plays a critical role in sensing the nutrient status. The skeletal muscle stores many amino acids and is an important source of amino acids under conditions of amino acid deprivation [77]. Signaling starting from the umami receptor (T1R1/T1R3) may be involved in the physiology of skeletal muscle metabolism and pathology of skeletal muscle diseases through the regulation of autophagy. Further studies are required to confirm this relationship.

## 7. Factors Regulating Myogenesis and Transcription of MRFs

Several signaling pathways play essential roles in the behaviors of skeletal muscle stem cells. Notch signaling is a major regulatory pathway in muscle progenitor pools. Notch signaling has been shown to inhibit myoblast differentiation in several models [78,79,80,81,82,83,84,85,86,87,88]. Bone morphogenetic protein (BMP) signaling is also involved in maintaining the proliferative status of muscle progenitors [89]. Loss- and gain-of-BMP function experiments in the limbs of fetal chicks showed that activation of BMP signaling leads to an increased number of Pax7-positive cells and increased muscle mass. Inhibition of BMP signaling impairs the growth of skeletal muscle, suggesting that BMP promotes the proliferation of muscle progenitor cells [90]. In mice, BMP regulates postnatal growth and adult muscle homeostasis by regulating the proliferation of satellite cells, whereas BMP inhibition impairs muscle growth [91,92]. Moreover, an inhibitor of DNA-binding protein 1, a BMP target gene, is necessary to maintain muscle satellite cells in an undifferentiated or proliferative status [93,94]. In an experiment using C2C12 cells, BMP induced osteoblast differentiation while suppressing myogenic differentiation [95]. Smad4, a downstream effector of BMP-Smad (canonical BMP) signaling, is a critical suppressor of BMP in myogenic differentiation [96].

The demand for Wnt signaling during myogenesis differs between the fetal and embryonic phases [97]. Embryonic myoblasts in the developing limbs of mice normally form progenitors and skeletal muscle fibers. In the fetal stages, progenitors that express a constitutively active form of β-catenin, an effector molecule of canonical Wnt signaling, increase the number of Pax7-positive cells [97]. Wnt7a enhances the symmetrical division of satellite cells during skeletal muscle regeneration by maintaining the stem cell pool [98]. Further studies reinforced the importance of BMP and Wnt signaling in myogenesis. Recapitulated myogenesis of pluripotent stem cells was achieved through the action of specific molecules at precise time points. When epiblast-like cells were treated with a BMP inhibitor (LDN193189) and Wnt activator (CHIR99021), commitment of the presomitic mesoderm was initiated [99]. Following the addition of a growth factor cocktail (fibroblast growth factor 2, hepatocyte growth factor, and insulin-like growth factor 1), the cell population displays a mixture of differentiated myocytes (myogenin-positive) and progenitor cells (Pax7-positive) [100,101]. Taken together, many growth factors and signaling molecules are involved in regulating the myogenesis and transcription of MRFs. Moreover, these factors and signals may be involved in cross-talk.

## 8. TLE3 Represses Transcription of MyoD and Suppresses Myogenesis

**Figure 4:**
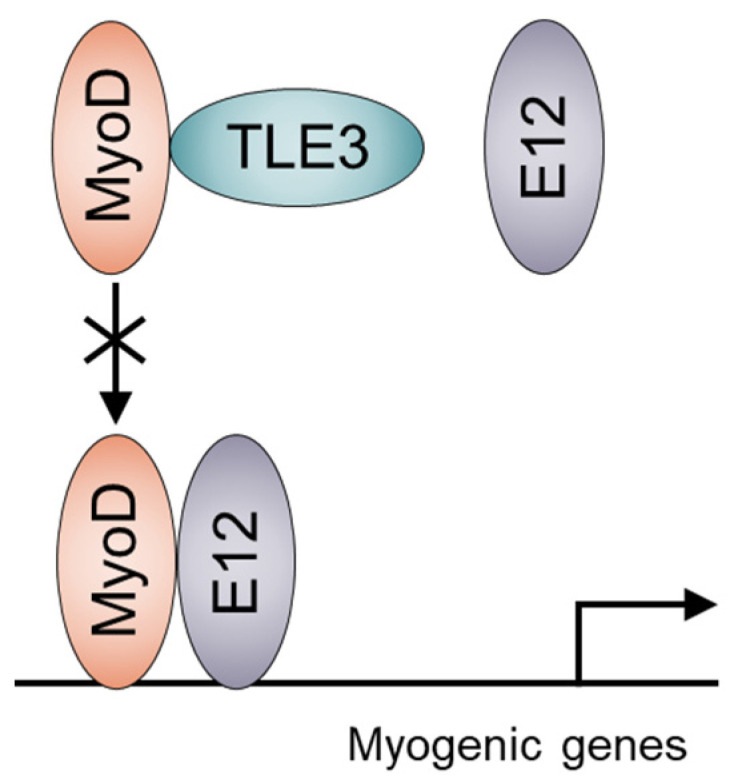
Model for transcriptional regulation of MyoD by the transcription cofactor, TLE3.

Groucho/TLE proteins are transcriptional cofactors that do not directly bind to DNA but play essential roles in cell differentiation [102]. Members of the Groucho/TLE family, including TLE3, suppress Wnt-β-catenin signaling as transcriptional co-repressors of Tcf/Lef, which are downstream effectors of Wnt-β-catenin signaling and subsequently repress transactivation of the Wnt target gene [102,103,104,105]. We previously identified Tcf/Lef-responsive elements in the 5′ untranslated regions of TLE3 through comparative genomic and functional analyses. We demonstrated that Wnt-β-catenin signaling directly increases the expression of TLE3 in W20-17 cells, a murine bone marrow mesenchymal stem cell line [106]. Groucho/TLE family proteins are composed of a five-domain structure [107]: the Q domain, which is glutamine-rich and highly conserved, forms two coiled-coil motifs that can oligomerize [108,109,110]; a glycine/proline-rich domain, which is critical for recruiting and interacting with histone deacetylases [109,111,112]; a CcN domain, which contains putative phosphorylation sites of cdc2 and casein kinase II and a nuclear localization sequence; a serine/proline-rich domain, which contains a region enriched by serine/proline [107,113,114,115]; and a WD40 domain, which is highly conserved and contains tandem repeats of tryptophan and aspartic acid. X-ray crystallography revealed that the WD40 domain forms a β-propeller and binds various transcription factors [102]. TLE3 is expressed in the myoblasts of murine embryos, and its expression overlaps with that of both MyoD and Myf5 [116]. TLE3 is also expressed in cells with the same mesenchymal lineage as myoblasts, including bone marrow mesenchymal stem cells [117,118,119], myoblasts, adipocytes, and osteoblasts. MyoD is a master transcription factor of myogenesis, and PPARγ and Runx2 are master transcription regulators of adipogenesis and osteoblastogenesis, respectively [120,121]. Villanueva et al. [122] identified TLE3 as a transcriptional coactivator of PPARγ in adipocytes, and it was later confirmed that TLE3 enhances the transcriptional activation of PPARγ and induces adipogenesis of bone marrow mesenchymal stem cells [118,119]. Interference with the interaction between PPARγ and PRDM16 causes TLE3 to shift the fate of adipose stem cells from brown fat cells to white fat cells, thus impairing fatty acid oxidation and thermogenesis [123]. Endogenous TLE3 interacts with MyoD in C2C12 cells. TLE suppresses myoblast differentiation in C2C12 cells and is induced by overexpression of MyoD in C3H10T1/2 cells [124]. To confirm whether the suppressive effect of TLE3 on myogenesis is due to repression of MyoD-dependent transactivation, we employed luciferase reporters driven by muscle creatine kinase (MCK0.8) and myogenin (MG185) promoters. TLE3 suppresses the transcriptional activation of MyoD and other MRFs including MRF4, Myf5, and myogenin. Moreover, ChIP assays showed that TLE3 disrupts the binding of MyoD to the promoter region of myogenin [124]. Typically, the WD domain of Groucho/TLE plays a critical role in transcriptional regulation [125]. We previously showed that the WD domain of TLE3 is necessary to repress the transcriptional activity of Runx2. However, the WD domain-deletion mutant still represses the transcriptional activity of MyoD, and both the Q and serine/proline-rich domains of TLE3 are essential for interacting with and repressing MyoD [124]. MRFs interact with E proteins, including E12, through their bHLH domains [126]. TLE3 also interacts with the bHLH domain of MyoD. Although overexpression of E12 dose-dependently counters the suppressive effect of TLE3 on transactivation of MyoD activity, TLE3 does not interact with E12 but rather disrupts the interaction between MyoD and E12. This result suggests that TLE3 masks the E protein-binding region on MyoD; thus, TLE3 competitively interferes with MRF and E protein interactions [124] (Figure 4). Together, TLE3 facilitates the differentiation of mesenchymal stem cells into the white adipocyte lineage by activating PPARγ, an adipogenic master regulator, and suppresses the transcription of Runx2 and MyoD, which are master regulators of osteoblastogenesis and myogenesis, respectively. The role of TLE3 in skeletal muscle in vivo remains unknown. Studies are needed to analyze muscle-specific TLE3 knockout mice because TLE3 null mice are embryonically lethal.

## 9. NF-κB Represses Transcription of MyoD and Suppresses Myogenesis

NF-κB is a transcription factor that is closely related to various physiological and pathological conditions, such as immune system, cell survival, and inflammatory processes. In response to stimuli such as tumor necrosis factor (TNF)-α, the inhibitor of κBα is degraded, and p65, the main subunit of NF-κB, forms a complex with p50. This heterodimer complex of p50/p65 is translocated from the cytoplasm to the nucleus. In the nucleus, the p50/p65 heterodimer binds to the responsive elements of NF-κB to regulate target gene expression [127]. In a mouse model, the regeneration of skeletal muscle following trauma was improved following systemic administration of the NF-κB inhibitor curcumin [128]. Several groups reported that activation of NF-κB suppresses the differentiation of skeletal muscle by reducing MyoD protein levels [129,130,131,132]. In contrast, NF-κB signaling is necessary for myoblast proliferation [129,130,131,132]. Because MyoD regulates the exit from the cell cycle, in part through transactivation of the cell cycle inhibitor p21, NF-κB suppresses myoblast differentiation through the sustained proliferation of myoblasts [129,133]. In addition, NF-κB mediates the suppression of myoblast differentiation by inducing cyclin D1, a member of the cyclin family of proteins, which increases cell cycle progression from the G1 to S phase [129]. We reported that treatment with a TNF-α ligand suppresses MG185-luciferase activity induced by overexpression of MyoD in a TNF-α concentration-dependent manner in vitro. To confirm this function, we generated C-terminal-truncated constructs of p65, the main subunit of NF-κB and downstream effector of TNF-α. p65 consists of transactivation domain 1, transactivation domain 2 (TA2), Rel homology domain, DNA-binding domain, and dimerization domain. Although the full-length and transactivation domain 1 domain-deleted p65 mutant suppressed the MG185-luciferase activity induced by MyoD induction, the TA2 domain-deleted mutant p65 did not. These results indicate that TNF-α suppresses transactivation of MyoD via the canonical NF-κB signaling effector p65-TA2 domain [132]. The DNA binding capacity and transcriptional activation of NF-κB decrease during myoblast differentiation [129,134]. Insulin-like growth factors stimulate the activity of NF-κB during myogenesis [135,136]. We previously determined the activity of NF-κB during skeletal muscle regeneration in a mouse model. NF-κB signaling was active from days 1 to 3 after injection of cardiotoxin, which corresponded to the proliferation stage of satellite cells. This behavior of NF-κB signaling was similar to the expression levels of cyclin A2 and was related to the progression of the S phase in the cell cycle [137]. NF-κB activation occurs prior to the increase in myogenin levels [132]. However, the role of NF-κB (p65) in the skeletal muscle in vivo remains unknown. Further studies of muscle-specific p65 knockout mice are needed because p65-null mice are embryonically lethal.

## 10. Conclusions

Maintaining skeletal muscle mass and function is important for a healthy lifestyle. Elderly populations in both developed and developing countries have increased, and strategies for regenerating and restoring skeletal muscle mass and function are urgently needed. Therefore, the research focus has rapidly shifted from the development of muscle to the regenerative medicine of skeletal muscle tissue, particularly using stem cells. Researchers have attempted to modify the expression of genes essential for generating muscles in vivo to recapitulate myogenesis ex vivo. In vitro myogenesis protocols are currently used to model muscle-associated diseases in vitro for drug discovery or cell therapy, in which functional muscles are produced in the laboratory for tissue replacement therapy. In this review, we highlight the function of MRFs and their modulators, such as TLE3 and NF-κB, and target genes, such as taste receptors. Further research is needed to understand the function of MRFs and the physiology or pathology of satellite cells, which will contribute to the development of cell therapies or drug discovery for muscle-related diseases.

## Data Availability

Not applicable.
